# Static Stretching as a Multilevel Mechanobiological Process: An Integrative Narrative Review and Proposed Framework from Mechanical Loading to Biological Adaptation

**DOI:** 10.3390/jfmk11030365

**Published:** 2026-09-10

**Authors:** Galina Nikolaeva Rusimova

**Affiliations:** Department of Anatomy and Biomechanics, National Sports Academy “Vassil Levski”, 1700 Sofia, Bulgaria; galina.rusimova@nsa.bg

**Keywords:** static stretching, mechanobiology, mechanotransduction, fascia, extracellular matrix, connective tissue, flexibility

## Abstract

Static stretching is one of the most widely prescribed interventions in sports medicine, rehabilitation, and exercise science for improving joint range of motion (ROM) and flexibility. Despite decades of research, however, the biological mechanisms underlying its effects remain incompletely understood. Traditional explanations have primarily focused on muscle extensibility and passive tissue stiffness, whereas emerging evidence suggests that stretching may engage multiple mechanically and biologically interconnected systems. This integrative narrative review aimed to synthesize current evidence on the biological mechanisms underlying static stretching and to propose a conceptual, hypothesis-generating framework linking externally applied mechanical loading with biological responses across multiple levels of organization. Evidence from biomechanics, fascial biology, mechanotransduction, connective tissue biology, vascular physiology, and neuroscience was critically integrated, while distinguishing direct human static-stretching evidence from indirect human, preclinical, and general mechanobiological evidence. Although evidence supports contributions from several of these individual domains, direct human evidence demonstrating their integrated operation during static stretching remains limited. The available evidence therefore suggests that static stretching may be understood more broadly than as a muscle-centered flexibility intervention alone. Mechanical loading during stretching may be transmitted across muscular and connective tissues and interact with mechanosensitive cellular, extracellular-matrix, vascular, inflammatory, and neural processes; however, the directness and strength of evidence for these proposed links vary substantially. The resulting framework provides a plausible, testable hypothesis for how interactions among these processes may contribute to heterogeneous findings and inter-individual variability following stretching interventions, rather than an established explanatory model. Accordingly, this review conceptualizes static stretching as a biologically active mechanical stimulus whose effects may involve interacting responses across multiple levels of biological organization. By integrating evidence from previously separated biological domains while explicitly recognizing differences in evidence directness, the proposed framework provides a theoretical basis for future mechanistic research and for testing the causal and temporal relationships among these candidate processes in humans.

## 1. Introduction

Static stretching remains one of the most widely used interventions in sports medicine, rehabilitation, and exercise science, with consistent evidence supporting its effectiveness for increasing joint range of motion (ROM) and flexibility [1,2,3]. It is also commonly incorporated into warm-up routines, injury-prevention programs, and rehabilitation protocols across physically active and clinical populations [2,4,5,6]. However, its effects on physical performance are context-dependent and may vary according to stretching duration, intensity, timing, population characteristics, and task demands [5,6]. Evidence regarding injury prevention is considerably more limited and remains inconclusive [2,6,7]. Despite its widespread application and decades of research, the biological mechanisms underlying static stretching remain incompletely understood, and considerable debate persists regarding the relative contributions of different tissues and processes to acute and long-term responses [3,8]. Emerging evidence, together with established principles from mechanobiology, suggests that these responses may involve interactions among muscular and fascial tissues, mechanotransduction, extracellular matrix processes, cellular signaling, and neural regulation rather than changes in muscle mechanical properties alone [9,10,11,12].

Previous reviews have substantially advanced understanding of static stretching by examining outcomes such as flexibility and ROM, muscle strength and power, physical performance, and injury-related outcomes [5,6,7,13,14]. Other integrative contributions have extended this perspective beyond muscle-centered explanations. Stecco et al. [10] examined the mechanical contributions of muscle, tendon, and fascia during passive stretching and emphasized their integration within the myofascial unit; Zvetkova et al. [12] reviewed biomechanical, physiological, healing, and therapeutic effects of stretching, including connective-tissue processes; Pirri et al. [15] characterized fascia as a mechanosensitive tissue and regenerative niche involved in extracellular matrix remodeling and immune–vascular regulation; and Stecco et al. [16] integrated muscle, connective tissue, neural, and vascular components within the concept of the myofascial unit. Collectively, these contributions provide an important foundation for a broader biological interpretation of stretching and myofascial function.

Building on this literature, the present review adopts a complementary cross-level perspective specifically centered on static stretching as a mechanical stimulus. Rather than proposing that the individual mechanisms considered here are novel, the proposed framework integrates evidence across interacting levels of biological organization—from tissue-level mechanical responses and myofascial force transmission to cellular mechanotransduction, extracellular matrix processes, vascular and inflammatory responses, and neural regulation. Importantly, the strength and directness of evidence are not equivalent across these levels: some mechanisms are supported by direct human stretching studies, whereas others are informed primarily by indirect evidence from human studies, preclinical research, or general mechanobiological evidence. Accordingly, the proposed framework should be regarded as an evidence-informed, hypothesis-generating model rather than as an established causal sequence.

From this perspective, static stretching provides a useful model for considering how externally applied mechanical loading may interact with multiple biological systems. Mechanical forces are distributed across musculotendinous and fascial tissues [10], while established principles of cellular mechanotransduction provide biologically plausible pathways through which mechanical stimuli can influence intracellular signaling, gene regulation, and extracellular matrix behavior [9,11,17,18]. However, much of the evidence defining these pathways derives from general mechanobiology rather than direct studies of static stretching in humans. Their specific contribution to stretching-induced adaptation therefore remains incompletely established. These mechanobiological principles provide a basis for examining how tissue-level mechanics may interface with extracellular matrix, vascular/inflammatory, and neural processes, while recognizing that the directness of evidence linking each of these domains to static stretching varies substantially.

The aim of the present integrative review is to synthesize current evidence relevant to the mechanobiological mechanisms of static stretching and to propose a cross-level conceptual framework linking mechanical loading with biological responses across tissue, cellular, extracellular, vascular/inflammatory, and neural domains. By distinguishing direct evidence from mechanistically plausible but less directly established pathways, the review seeks to clarify how current findings can be integrated, where causal inference remains limited, and which mechanistic relationships require further experimental investigation.

## 2. Literature Search and Review Approach

This article was designed as an integrative narrative review of the biological mechanisms underlying static stretching across multiple levels of organization. The review integrates evidence from biomechanics, mechanobiology, connective-tissue biology, neurophysiology, vascular biology, and exercise science. To improve methodological transparency and reduce confirmation and citation-selection bias, literature identification and study selection followed explicit scope and eligibility criteria, with evidence subsequently mapped according to its relevance and directness to human static stretching.

Structured searches were conducted in PubMed, Scopus, and Web of Science Core Collection across six predefined mechanistic domains: (1) tissue mechanics and muscle–tendon–fascia properties; (2) mechanotransduction and cellular signaling; (3) extracellular matrix remodeling; (4) vascular mechanobiology; (5) inflammatory mechanobiology and tissue repair; and (6) neural adaptations and stretch tolerance. Each search combined “static stretching” OR “static stretch” with domain-specific concept blocks. PubMed was searched on 22–23 August 2026, Scopus on 23 August 2026, and Web of Science on 24 August 2026 (final search date). No publication-date or language restrictions were applied at the search stage. The searches yielded 480 PubMed, 683 Scopus, and 802 Web of Science records (1965 raw retrievals before deduplication). Complete database-specific strategies, fields, limits, dates, and retrieval counts are provided in Supplementary Table S1.

Eligibility was determined by the predefined scope rather than compatibility with the proposed framework. Direct human studies were eligible when they investigated static stretching and reported a mechanistic outcome within at least one of the six domains. Indirect human, animal, ex vivo, in vitro, or cellular studies were eligible as supporting mechanistic evidence when the model or mechanical stimulus was relevant to a candidate mechanism, but were not treated as evidence that the same mechanism occurs during human static stretching. Eligible outcomes included stretch tolerance/ROM, tissue stiffness, architecture or deformation, mechanotransduction/signaling, extracellular-matrix remodeling, vascular responses, inflammatory/tissue-repair responses, and neural/reflex/sensory outcomes. Studies were excluded for absence of relevant mechanistic evidence, inseparable non-static stretching interventions, performance or clinical outcomes without a mechanistic outcome, clearly irrelevant models or stimuli, insufficient methodological information, retraction, or duplicate reporting. Supportive, null, mixed, and conflicting findings remained eligible; compatibility with the framework was not an inclusion criterion.

After consolidation, duplicates were removed primarily by DOI/PMID and subsequently by title, author, and year. Of 1965 raw retrievals, 1198 duplicate or overlapping records were removed, leaving 767 unique records for single-reviewer title/abstract screening as Include, Exclude, or Uncertain. Of 83 initially uncertain records, 43 were subsequently included and 40 excluded; overall, 245 records were excluded and 522 retained. Full-text information was consulted when eligibility or interpretation could not be established reliably from bibliographic information and the abstract. Report-level reconciliation of the 522 retained records yielded 514 unique database-derived studies.

The database-derived evidence set was then supplemented by bibliographic reconciliation and verification of sources identified through manual, reference-chaining, contextual, and foundational routes. A total of 39 additional references were individually assessed rather than automatically incorporated: 9 met the eligibility criteria as supporting mechanistic evidence, 29 were retained separately as contextual/foundational sources, and 1 was classified as a bibliographic/QC item. The resulting evidence map therefore comprised 523 study-level or supporting evidence records (514 database-derived studies plus 9 additional eligible sources); contextual/foundational sources were not counted as study-level evidence supporting specific mechanistic claims regarding human static stretching.

The 523 records were organized in Supplementary Table S2, which documents bibliographic information, mechanistic domain, model/population, intervention or stimulus, outcome/mechanistic level, main finding, evidence directness, source/study type, QC status, identification route, and extraction-source information. Evidence was differentiated as direct human, indirect human or indirect/contextual human, indirect/preclinical, review/contextual synthesis, or general mechanobiological/theoretical evidence according to its relationship to mechanistic claims about human static stretching. Direct human evidence received the greatest interpretative weight for such claims; indirect and preclinical evidence supported biological plausibility, while review/contextual and theoretical evidence supported synthesis and context rather than mechanism-specific causal inference.

Supportive, null, mixed, and conflicting evidence was retained in the synthesis, with disagreements interpreted in relation to population/model, stretching exposure, outcome, and major methodological limitations. Directness and methodological quality were considered separately. Where evidence was indirect, inconsistent, or methodologically limited, claims were qualified as proposed, plausible, or indirectly supported rather than established. The framework was therefore revised in light of the screened evidence rather than used to determine evidence inclusion.

Reference and evidence-map verification used a risk-based quality-control procedure covering manually identified supporting sources, previously integrity-corrected and limited-access records, and stratified samples of direct-human, review/contextual, and preclinical evidence. Database-derived records were checked against available source and bibliographic information, and manually identified supporting sources against PubMed or publisher records. Conservative descriptors were retained when source information did not support greater specificity. No numerical study-quality score or formal risk-of-bias grading system was applied.

No original statistical analysis or quantitative evidence synthesis was performed. Where published meta-analytic findings were used quantitatively, effect estimates and corresponding confidence intervals were reported when relevant and available, without interpreting statistical significance as evidence of mechanistic dominance.

## 3. Evolution of the Static Stretching Concept

### 3.1. Early Concepts of Static Stretching

Static stretching has long been used in rehabilitation, physical activity, and sport as a practical intervention for increasing flexibility and joint range of motion (ROM). Earlier clinical and exercise-oriented accounts commonly emphasized muscle or musculotendinous extensibility in explaining stretching-induced changes in flexibility [4,8]. Subsequent evidence has broadened this interpretation. Contemporary systematic and meta-analytic evidence indicates that improvements in ROM following static stretching may involve changes in mechanical properties as well as sensory factors such as stretch tolerance, rather than requiring a simple structural elongation of muscle [1,3].

The evolution of mechanistic thinking is therefore better characterized as a progressive broadening of predominantly musculotendinous explanations rather than replacement of a “muscle-only” model by a “fascial” model. Current interpretations consider potential contributions from muscle, tendon, connective tissue, neural regulation, sensation, and joint-specific factors, with the relative contribution of these determinants varying according to the stretching exposure, outcome, and experimental context [3,8,10]. Evidence supporting fascial involvement expands this multifactorial perspective but does not establish fascia as the dominant target of static stretching [10].

### 3.2. The Muscle–Tendon Paradigm

Musculotendinous mechanical explanations have historically played a central role in the interpretation of stretching-induced changes in flexibility. Within this perspective, increases in joint range of motion (ROM) were commonly attributed to greater extensibility and altered passive mechanical properties of the muscle–tendon unit. These concepts provided a useful biomechanical basis for understanding stretching, but they did not necessarily imply that skeletal muscle was the only structure involved or that structural elongation represented the sole mechanism underlying improvements in ROM [4,8]. Contemporary evidence confirms that static stretching can increase ROM [1], while indicating that the mechanisms responsible for these changes are more heterogeneous than a simple muscle-lengthening model would predict.

Recent quantitative evidence illustrates this complexity. In a systematic review, meta-analysis, and multivariate meta-regression, Ingram et al. [3] reported small reductions in overall stiffness following both acute static stretching (Hedges’ g = 0.42, 95% CI 0.21–0.63) and chronic static stretching (g = 0.37, 95% CI 0.18–0.56). Chronic stretching also produced a moderate increase in maximum tolerable passive resistive torque, used as an indicator of stretch tolerance (g = 0.74, 95% CI 0.38–1.10), whereas neither acute nor chronic stretching produced a significant overall change in fascicle length. Importantly, chronic improvements in ROM were associated with both decreased overall stiffness (g = 0.59, 95% CI 0.08–1.10) and increased stretch tolerance (g = 0.74, 95% CI 0.41–1.09), suggesting that mechanical and sensory adaptations may contribute concurrently rather than representing mutually exclusive mechanisms. However, the certainty of evidence for the mechanistic outcomes assessed in this meta-analysis was rated as low or very low, precluding conclusions regarding the dominance of any single mechanism [3].

A similarly nuanced interpretation applies to the broader assumptions historically associated with stretching. Evidence concerning physical performance indicates that responses depend on factors including stretching duration, intensity, timing, and whether acute or repeated exposure is considered [5,6,14]. Likewise, current evidence does not support static stretching as a universally effective strategy for injury prevention, and its relevance depends on the activity, injury type, stretching protocol, and broader training context [6,7]. These findings have progressively expanded the musculotendinous perspective rather than simply replacing it. Muscle and tendon mechanical properties remain important components of the response to static stretching, but contemporary interpretations increasingly incorporate stretch tolerance, neural and sensory regulation, connective-tissue contributions, and joint- and protocol-specific factors. The development of the field is therefore better characterized as a transition toward a multifactorial model than as replacement of a muscle–tendon paradigm by a fascia-centered one.

### 3.3. Emergence of the Myofascial Concept

Growing interest in connective-tissue biomechanics has broadened the anatomical interpretation of stretching beyond the musculotendinous unit alone. Skeletal muscle is increasingly considered within an integrated myofascial system in which muscle, tendon, fascia, and surrounding connective tissues can interact mechanically and contribute to force transmission and load distribution [10,16]. This perspective does not imply that fascia is the dominant mechanical target of static stretching in all contexts; rather, it identifies fascial tissues as one potential contributor within a multicomponent system.

Evidence supporting this broader interpretation comes from several levels. Review and modeling studies indicate that epimuscular myofascial connections can influence force transmission and muscle mechanics, while anatomical and biomechanical syntheses support continuity and mechanical interaction across fascial structures. More directly, a randomized crossover study in humans found that a 5 min static plantar-flexor stretch reduced stiffness in both skeletal muscle and deep fascia, with the reduction in fascial stiffness showing a small association with the increase in ankle ROM [19]. These findings support fascial involvement in the mechanical response to stretching, but they do not establish fascia as the principal limiter of ROM or as the dominant target across anatomical regions, stretching protocols, or individuals.

The myofascial concept has also provided a broader framework for considering how mechanically interconnected tissues may interface with cellular and extracellular processes. Fascia is increasingly recognized as biologically active and mechanosensitive, and review-level mechanobiological evidence links fascial tissues with mechanotransduction, extracellular-matrix regulation, and neurovascular interactions [15,16]. However, much of this evidence derives from general mechanobiology, review synthesis, modeling, or preclinical work rather than direct demonstration during human static stretching. Accordingly, the myofascial perspective is best understood as an expansion of the multifactorial model of stretching rather than a replacement of the musculotendinous model by a fascia-centered paradigm.

### 3.4. Toward an Integrated Mechanobiological Model

The evolution of stretching research has progressively broadened the interpretation of static stretching beyond a predominantly muscle-centered intervention. Contemporary evidence indicates that improvements in ROM may involve changes in passive mechanical properties together with sensory adaptations such as increased stretch tolerance, rather than requiring structural muscle elongation alone [3]. In parallel, advances in myofascial research have provided a broader anatomical framework in which muscle, fascia, and associated connective tissues are considered mechanically interconnected rather than functionally isolated [10,16]. This conceptual broadening provides a basis for investigating the mechanobiological processes that may contribute to stretching-induced adaptation.

The emerging model also highlights the contribution of neural and sensory factors to flexibility. However, muscular, connective-tissue, neural, and sensory determinants of ROM are not readily separable in vivo and may contribute concurrently to the response to static stretching. Contemporary evidence indicates that improvements in ROM can be associated with both changes in passive mechanical properties and increased stretch tolerance, while direct human experiments demonstrate that neural and mechanical responses may occur simultaneously and vary according to characteristics of the stretching stimulus [3,20]. Accordingly, interindividual differences in responses to static stretching should not be interpreted as evidence that ROM is determined by a single identifiable limiting structure. Rather, they support a multifactorial perspective in which interacting mechanical, neural, and sensory processes may contribute with differing relative importance across individuals and experimental conditions [3,20].

Accordingly, contemporary evidence indicates that static stretching should not be interpreted solely as a technique for increasing muscle length through mechanical elongation of the musculotendinous unit. Instead, changes in ROM may reflect contributions from passive mechanical properties and sensory adaptations such as stretch tolerance, while muscular, connective-tissue, neural, and sensory processes provide a broader context in which these responses can be interpreted [1,3,10,16]. The relative contribution of these factors remains incompletely resolved and may vary according to the stretching protocol, anatomical region, outcome assessed, and individual characteristics [1,3].

Recent studies further demonstrate that prolonged, high-volume static stretching may induce not only improvements in flexibility but also neuromuscular and morphological adaptations, including increases in muscle strength and hypertrophy. Importantly, these findings derive from prolonged and unusually high-volume stretching protocols and should not be generalized to conventional short-duration stretching prescriptions. For example, Warneke et al. employed 1 h of daily plantar-flexor static stretching for 6 weeks and reported increases in maximal voluntary contraction, muscle thickness, and flexibility [21]. These findings emphasize that the physiological effects of static stretching depend on the intensity, duration, frequency, and cumulative dosage of the applied mechanical stimulus, supporting the view that stretching should be regarded as a dose-dependent biological intervention rather than a uniform flexibility technique [14,21].

Collectively, these developments have redirected scientific attention toward the biological mechanisms through which mechanical loading is sensed, transmitted, and translated into tissue adaptation [9,10,11,12,17,18]. This conceptual evolution provides the foundation for the following sections, which examine mechanotransduction, extracellular matrix remodeling, vascular and inflammatory responses, and neuromuscular regulation as potentially interacting processes relevant to adaptation to static stretching [9,10,11,12,17,18]. Within this framework, the myofascial system provides an interconnected anatomical context through which mechanical forces may be transmitted across muscular and connective tissues [10,16].

The traditional muscle-centered paradigm (left) conceptualizes static stretching as an intervention that improves joint range of motion (ROM) primarily through increased muscle extensibility. In contrast, the contemporary multi-tissue paradigm (right) views static stretching as a mechanical stimulus acting on an integrated multi-tissue system, where mechanical loading is distributed across interconnected tissues and may initiate mechanobiological processes that contribute to changes in tissue properties and joint range of motion. 

## 4. Fascia in the Mechanical Response to Static Stretching

### 4.1. Broadening the Musculotendinous Perspective

Traditionally, static stretching has been interpreted predominantly within a musculotendinous framework, with improvements in flexibility commonly attributed to increased muscle or musculotendinous extensibility [8]. These concepts provided an important basis for understanding stretching-induced changes in ROM, although they did not exclude contributions from other tissues or sensory mechanisms.

Advances in anatomy, biomechanics, connective tissue biology, and fascial research have broadened this interpretation. Contemporary evidence indicates that muscles, tendons, and fascia function as components of an integrated myofascial unit rather than isolated anatomical structures [10,16]. This concept has recently been expanded by redefining the fascial system as a body-wide, multiscale connective tissue network integrating musculoskeletal, neural, visceral, and superficial fascial components into a continuous anatomical and functional system capable of transmitting mechanical loads throughout the body [22]. 

This evolving perspective is supported by anatomical and biomechanical evidence demonstrating that fascia forms a continuous three-dimensional network linking muscles, tendons, ligaments, bones, and neurovascular structures. Rather than acting as isolated connective tissue layers, these interconnected tissues contribute to force transmission, proprioception, mechanical load distribution, and coordinated mechanical behavior throughout the musculoskeletal system [10,22,23].

The functional significance of fascia extends beyond its anatomical organization. Experimental, anatomical, and modeling evidence indicates that deep fascia and epimuscular myofascial connections may contribute to force transmission and influence muscle mechanics under specific loading conditions, although precise in vivo quantification remains challenging [10,16,23]. Consequently, flexibility and joint ROM may reflect contributions from multiple mechanically and functionally interconnected tissues rather than skeletal muscle alone [10,16].

This conceptual broadening has important implications for understanding the biological mechanisms underlying static stretching. If muscles, fascia, and other connective tissues operate as mechanically interconnected components, stretching-induced responses are unlikely to be confined to muscle fibers alone. Instead, static stretching can be viewed as a mechanical stimulus acting across interconnected tissues, with fascial structures representing one potential contributor to tissue deformation, mechanosensation, and force transmission within this broader system. This perspective provides the conceptual foundation for the mechanobiological mechanisms discussed in the following sections.

### 4.2. Fascia as a Potential Contributor to the Mechanical Response to Static Stretching

**Figure 1:**
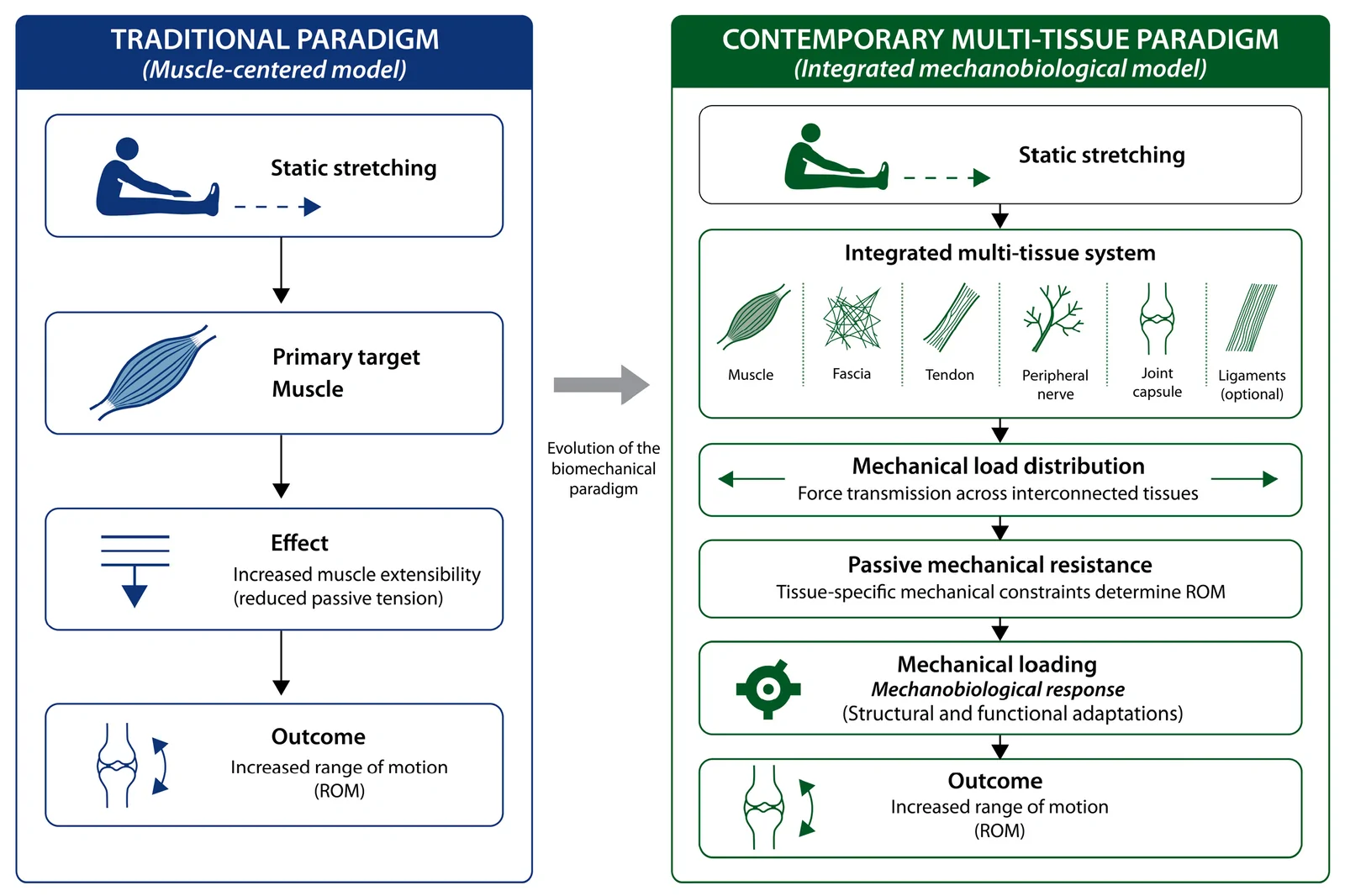
Evolution of the conceptual paradigm of static stretching from the traditional muscle-centered model to a contemporary integrated multi-tissue and mechanobiological model.

As illustrated in Figure 1, contemporary evidence suggests that passive joint range of motion (ROM) is an emergent property of the integrated myofascial system rather than skeletal muscle alone. This perspective has shifted one of the central questions in stretching research from whether muscle elongates during stretching to how multiple biological structures contribute to the mechanical response to passive loading [10,22,23]. Passive resistance during stretching arises from the combined mechanical behavior of several interconnected tissues, including skeletal muscles, tendons, fascia, peripheral nerves, joint capsules, and ligaments. These structures interact in complex series and parallel arrangements, while the measured mechanical response is also influenced by factors such as joint geometry, muscle activation, sensory tolerance, testing position, and measurement technique. Accordingly, a single tissue cannot necessarily be identified as reaching an independently identifiable mechanical limit. Consequently, the relative contribution of different structures to ROM may vary not only between individuals but also between joints within the same individual, indicating that flexibility cannot be explained solely by muscle extensibility [10,23,24].

Among these tissues, fascia has emerged as a potential contributor to the mechanical response to static stretching. Stecco et al. suggested that the deep fascia should be regarded as an active load-bearing component of the myofascial system rather than passive connective tissue covering. Its continuous three-dimensional organization and collagen-rich architecture enable the development and distribution of tensile forces during passive elongation, supporting a potential contribution of fascia to passive resistance during stretching [10,23]. This interpretation is consistent with the work of Schleip et al., who described fascia as both a mechanosensitive and force-transmitting tissue, extending its role beyond structural support [25]. Likewise, Wilke et al. reviewed evidence indicating that fascial continuity allows mechanical force transmission not only within individual muscles but also between neighboring and functionally related muscles, suggesting that stretching one anatomical region may influence distant body segments through myofascial connections [26]. The review and modelling evidence presented by Yucesoy and Huijing further supports the concept of epimuscular myofascial force transmission through connective tissue pathways [27].

Together, these findings challenge the classical assumption that skeletal muscle represents the sole mechanical target of static stretching [10,22,23]. Instead, passive stretching should be viewed as loading an integrated system of tissues in which the mechanical response depends on their structural organization and material properties rather than muscle architecture alone [10,23,25,26].

The mechanical behavior of fascia further reinforces this interpretation. Like other collagen-rich connective tissues, fascia exhibits anisotropic mechanical behavior during tensile loading, with mechanical properties that may vary according to tissue region and loading direction [28]. These findings indicate that fascial tissues can contribute to the mechanical response to tensile loading, although evidence from ex vivo thoracolumbar fascia cannot be directly generalized to the behavior of fascia during static stretching in vivo [28]. Consequently, the mechanical properties of fascia may contribute to the resistance experienced during stretching alongside those of muscle and other connective tissues [10,28].

Collagen architecture may therefore contribute to the mechanical properties relevant to flexibility [10,28]. The deep fascia consists predominantly of densely organized type I collagen fibers embedded within an extracellular matrix rich in elastin, proteoglycans, and hyaluronan [10]. This composition provides sufficient compliance to permit physiological movement while maintaining high tensile strength and efficient force transmission [10,25,28]. Consequently, differences in collagen organization, tissue hydration, and extracellular matrix composition may influence connective-tissue mechanical properties, although their specific contribution to passive ROM during static stretching remains uncertain [10,28].

Direct human experimental evidence provides further support for a potential contribution of fascia to the acute mechanical response to static stretching. Warneke et al. demonstrated that 5 min of static plantar-flexor stretching acutely reduced both muscle and deep-fascia stiffness, whereas dynamic stretching did not significantly reduce stiffness compared with the control condition [19]. Furthermore, the reduction in deep-fascia stiffness showed a small association with the increase in ankle ROM [19]. These findings suggest that deep fascia may contribute to the acute mechanical response to static stretching, although they do not establish fascia as the primary determinant of stretching-induced increases in ROM [10,19].

Collectively, current evidence indicates that passive ROM emerges from the mechanical interaction of muscles, fascia, tendons, peripheral nerves, and other connective tissues rather than from muscle extensibility alone [10,22,23,25,26]. Within this integrated biomechanical model, fascia may contribute to passive stiffness and tensile-force transmission within the myofascial network and may respond to mechanical loading [10,22,23,25,28]. Interpretation of elastography-derived measures of fascial stiffness nevertheless requires caution. Strain-elastography measurements of fascial tissue show protocol- and observer-dependent reproducibility, whereas shear-wave elastography measurements may be influenced by acquisition depth and probe pressure [29,30]. Direct human evidence therefore supports a potential contribution of deep fascia to the acute mechanical response to static stretching, whereas evidence for broader fascial force transmission and tissue-specific mechanical behavior remains largely indirect or contextual [19,23,25,26,28]. The relative contribution of fascia to ROM, its variability across anatomical regions and tissue depths, and its role in chronic adaptations remain uncertain.

### 4.3. Sources of Variability in Responses to Static Stretching

Although static stretching consistently increases ROM, the magnitude of response and the underlying mechanisms vary considerably across studies and individuals [1,2,3,6]. Potential sources of heterogeneity that should be considered include baseline ROM, stretch intensity and endpoint definition, total stretching dose, measurement error, sex and age, training status, familiarization, joint positioning, sensory tolerance, and intervention adherence; available systematic evidence indicates that the influence of individual moderators may differ between acute and chronic stretching responses [1,2,3]. Contemporary evidence, however, suggests an additional potential contributor to this variability: the involvement of non-muscular tissues in the mechanical response to stretching [8,10,24,26].

If fascia, peripheral nerves, or other connective tissues contribute to passive resistance during stretching, their tissue-specific mechanical properties may represent one potential source of variability in stretching responses [10,22,23,24,26,27]. Static stretching simultaneously loads muscles, fascia, tendons, peripheral nerves, and other connective tissues, each possessing distinct structural, viscoelastic, and physiological properties [10,22,23,25,26,27,28]. However, whether inter-individual differences in the relative contribution of these tissues directly explain heterogeneous responses to identical stretching protocols remains uncertain [8,24].

This interpretation is consistent with the concept of stretch tolerance. Weppler and Magnusson proposed that chronic improvements in ROM may often be explained by increased tolerance to the sensation of stretch rather than by structural changes in muscle tissue [8]. Nordez et al. further emphasized that non-muscular structures may contribute to the limitation of maximal joint ROM during stretching [24]. Together, these findings suggest that sensory and tissue-specific mechanical factors may both contribute to the inter-individual variability observed after similar stretching interventions [8,24].

Recent systematic evidence further supports the multifactorial nature of stretching adaptations. Takeuchi et al. reported that muscle–tendon unit stiffness decreases following acute static stretching, whereas long-term stretching showed only a tendency toward reduced stiffness [31]. Expanding these findings, Ingram et al. performed a systematic review, meta-analysis, and multivariate meta-regression examining changes in stiffness, stretch tolerance, and fascicle length associated with acute and chronic improvements in ROM [3]. Both acute and chronic static stretching were associated with reductions in overall stiffness, whereas increased stretch tolerance was identified following chronic but not acute stretching; evidence for changes in fascicle length was less consistent [3].

Additional evidence suggests that tissues other than skeletal muscle may contribute to the mechanical response during passive limb movement and stretching. Ridehalgh et al. demonstrated in vivo sciatic nerve excursion during passive limb movement, with movement patterns varying according to the testing condition, providing indirect human evidence that neural tissues participate mechanically during passive movement [32]. Similarly, Kalichman highlighted that myofascial continuity and tissue-specific mechanical behavior influence how tensile forces are distributed throughout the musculoskeletal system [23]. However, this evidence does not establish that neural or fascial tissues are the principal structures limiting ROM or that their mechanical behavior directly explains inter-individual variability in stretching responsiveness [23,32].

Direct human experimental evidence provides limited support for tissue-specific mechanical responses to acute static stretching. Warneke et al. demonstrated that 5 min of static plantar-flexor stretching acutely reduced both muscle and deep-fascia stiffness, with the reduction in deep-fascia stiffness showing a small association with the increase in ankle ROM [19]. Complementary systematic evidence in older adults indicates that acute static stretching can reduce passive muscle–tendon unit or muscle stiffness, although this does not establish that tissue-specific differences explain inter-individual variability in stretching responsiveness [33]. Thus, current evidence supports heterogeneity in the mechanical responses associated with stretching, but direct evidence that differences in the relative contribution of specific tissues account for heterogeneous responsiveness remains limited [19,33].

Collectively, current evidence indicates that stretching adaptations cannot be interpreted solely through the lens of muscle extensibility [3,8,10]. Instead, responsiveness to static stretching may be influenced by multiple interacting factors, including tissue-specific mechanical properties, neural regulation, stretch tolerance, and individual anatomical variability [1,2,3,8]. Direct human evidence demonstrates acute changes in muscle and deep-fascial stiffness following static stretching [19], systematic evidence indicates that changes in stiffness and stretch tolerance differ between acute and chronic ROM responses [3,31,33]. Evidence implicating fascia, peripheral nerves, or other non-muscular tissues as direct determinants of inter-individual stretching responsiveness remains largely indirect or contextual [23,24,25,26,27,32]. Thus, heterogeneous responses to static stretching are likely multifactorial, and the relative contribution of tissue-specific mechanical properties, sensory tolerance, participant characteristics, and intervention-related factors remains uncertain.

### 4.4. Fascial Contributions to the Mechanical and Biological Response to Static Stretching

The evidence presented in the preceding sections raises a fundamental question: which tissues contribute to the response to the mechanical stimulus imposed by static stretching? Although skeletal muscle has traditionally been regarded as the principal target of stretching interventions, current anatomical, biomechanical, and mechanobiological evidence suggests that this interpretation is incomplete [10,22,23,26]. Rather than acting exclusively on muscle fibers, static stretching distributes mechanical loading throughout the integrated myofascial system, with fascial tissues representing one of several connective-tissue components exposed to this mechanical stimulus [10,22,25].

This interpretation is supported by several complementary lines of evidence. Contemporary anatomical concepts describe the fascial system as a continuous, multiscale connective tissue network integrating musculoskeletal, neural, visceral, and superficial fascial components into a single functional system [22]. Within this network, fascia has been implicated in tensile-force transmission across adjacent and functionally related muscles [23,26], exhibits load-dependent mechanical behavior in ex vivo testing [28], and undergoes measurable acute changes in stiffness following static stretching [19]. Furthermore, improvements in joint range of motion are not consistently accompanied by proportional reductions in muscle stiffness, suggesting that changes in ROM cannot be attributed solely to changes in muscle stiffness [3,19,31].

An equally important argument is the biological responsiveness of fascial tissue. Schleip et al. proposed that fascia should be regarded as a mechanosensitive tissue rather than a passive connective structure because of its capacity to detect and respond to mechanical loading [25]. Supporting this concept, Langevin et al. demonstrated rapid cytoskeletal remodeling of subcutaneous tissue fibroblasts in response to mechanically induced tissue deformation, providing contextual cellular evidence for connective-tissue mechanosensitivity [34]. These observations provide a mechanobiological rationale for considering fascial and connective tissues as responsive to mechanical loading, but they do not establish that repeated static stretching induces chronic extracellular matrix remodeling in human fascia.

Although the molecular pathways through which connective tissues respond to mechanical loading are still being elucidated, Ingber proposed that mechanical loading is converted into biochemical activity through mechanotransduction, whereby physical forces regulate cellular behavior, gene expression, and extracellular matrix remodeling [9]. More recently, Pirri et al. further characterized fascia as a dynamic mechanobiological tissue involved in extracellular matrix remodeling, vascular regulation, immune signaling, and tissue regeneration [15]. Together, these findings provide a plausible mechanobiological framework through which mechanical loading could influence connective-tissue biology, but they do not by themselves establish long-term fascial adaptations to repeated static stretching. This framework provides a conceptual transition to the mechanotransduction mechanisms discussed in the following chapter.

Taken together, current evidence does not support replacing the traditional muscle-centered paradigm with an exclusively fascia-centered model. Instead, it supports an integrated mechanobiological framework in which fascia represents a potential contributor to the mechanical and biological response to static stretching, while functioning in continuous interaction with skeletal muscle, tendons, peripheral nerves, and the extracellular matrix. Within this perspective, static stretching is best understood as a mechanobiological stimulus that mechanically loads and may elicit biological responses across interconnected tissues of the myofascial system, rather than as an intervention acting on skeletal muscle alone.

### 4.5. Practical Implications

The emerging myofascial and mechanobiological perspective has important implications for future research and for the conceptual interpretation of stretching practice. Given the potential contribution of fascial tissues to passive mechanical resistance and joint range of motion (ROM), stretching interventions should not be considered under the assumption that muscle extensibility alone determines flexibility. Instead, static stretching should be regarded as a mechanobiological stimulus acting on an integrated myofascial system composed of skeletal muscle, fascia, tendons, peripheral nerves, and other connective tissues, each with distinct structural and mechanical characteristics and potentially different biological responses to loading.

From this perspective, the effectiveness of stretching interventions may be influenced not only by the stretching technique itself but also by the magnitude, duration, and frequency of mechanical loading, tissue-specific mechanical and biological characteristics, and individual mechanical constraints. Consequently, future research may investigate whether individualized approaches that consider multiple mechanical, sensory, and participant-specific factors can better account for variability in stretching responses than approaches assuming a uniform muscle-based constraint across individuals. However, such individualized or tissue-informed approaches remain prospective research hypotheses: the proposed tissue-specific determinants have not yet been validated as moderators of individual responsiveness, and no established clinical procedure currently exists for identifying a dominant tissue-level limitation and using it to guide stretching prescription.

Ultimately, recognizing the potential contribution of fascia within an integrated myofascial system provides a conceptual basis for investigating whether individualized and mechanistically informed stretching approaches can be developed and validated in future research, rather than supporting their current use as established clinical strategies.

## 5. Mechanotransduction

### 5.1. Mechanical Loading Initiates Cellular Signaling

Because static stretching mechanically loads multiple tissues within the integrated myofascial system, understanding how mechanical loading may be translated into biological responses requires consideration of mechanotransduction.

Mechanical deformation can function as a biologically active stimulus rather than merely producing passive tissue elongation. Externally applied tensile forces can be detected by cellular mechanosensors, including integrins, focal adhesion complexes, mechanosensitive ion channels such as PIEZO channels, and the cytoskeleton. These structures can convert mechanical deformation into biochemical signals involved in the regulation of gene expression, extracellular matrix remodeling, inflammatory signaling, and tissue adaptation [11,35,36,37]. However, direct evidence that conventional static stretching produces sufficient cellular strain in vivo to activate these specific mechanosensors and downstream pathways in human myofascial tissues remains limited.

Mechanotransduction is the fundamental biological process through which cells perceive and respond to changes in their mechanical environment. Contemporary mechanobiological research describes this process as a hierarchical cascade extending from membrane mechanosensing to cytoskeletal remodeling, nuclear deformation, and regulation of gene expression [9,11,17]. This multilevel integration provides a biological framework through which mechanical loading may influence cellular and tissue responses; however, evidence that transient mechanical loading during conventional static stretching induces sustained biological adaptations through these pathways in humans remains limited.

Mechanotransduction operates across multiple tissues and cell populations, although the specific responses depend on cell type, tissue context, and loading conditions [35,36]. Experimental evidence indicates that connective-tissue fibroblasts can respond directly to mechanical stretch through cytoskeletal remodeling and associated signaling mechanisms [38]. However, much of this evidence derives from cellular, ex vivo, or preclinical models rather than conventional static stretching in humans [38].

Collectively, current evidence supports mechanotransduction as a biologically plausible framework through which mechanical loading during static stretching may influence cellular responses across multiple tissues. However, direct demonstration of the activation of these pathways during conventional static stretching in humans, their dose–response relationships, and their causal contribution to ROM or clinical outcomes remains limited. This framework therefore provides a mechanistic basis for the candidate signaling pathways and tissue responses discussed in the following sections, while distinguishing biological plausibility from directly demonstrated effects of human static stretching.

### 5.2. Cellular Responses to Mechanical Loading

Following mechanosensing and signal transduction, mechanical loading can initiate cellular responses involved in tissue homeostasis and adaptation. These responses may involve multiple mechanosensitive cell populations and influence extracellular matrix (ECM) organization, cellular signaling, and tissue remodeling [35,36]. However, the extent to which these cellular responses are directly elicited by conventional static stretching in humans remains incompletely established.

Among these cells, fibroblasts are key mechanosensitive regulators of connective tissue adaptation. In response to mechanical loading, fibroblasts integrate physical cues transmitted through the extracellular matrix via interactions among integrins, focal adhesion complexes, the cytoskeleton, and intracellular signaling pathways [35,36]. These processes regulate cellular morphology, cytoskeletal organization, gene expression, collagen turnover, and extracellular matrix remodeling, thereby maintaining the structural and mechanical integrity of connective tissues. Experimental evidence further demonstrates rapid fibroblast cytoskeletal remodeling in response to static tissue stretch [38]. However, this evidence is indirect with respect to conventional human static stretching and does not establish chronic connective-tissue adaptation in humans.

The magnitude and direction of these adaptations depend on the characteristics of the applied mechanical stimulus. Loading magnitude, duration, frequency, and mode of application interact with tissue-specific mechanical properties to determine the extent of cellular responses [35,36]. Consequently, identical stretching protocols could elicit different cellular responses depending on the structural composition, mechanical environment, and biological state of the loaded tissue; however, dose–response relationships at the cellular level during conventional static stretching in humans remain insufficiently characterized.

Importantly, mechanobiological responses can involve crosstalk among multiple signaling pathways. Mechanical stimulation can influence vascular regulation, inflammatory activity, extracellular matrix turnover, tissue repair, and cellular homeostasis, with these processes potentially interacting within an integrated biological network rather than functioning as entirely independent biological events [35,36]. However, coordinated activation of these processes during conventional static stretching has not been directly demonstrated in humans.

Collectively, these findings support a mechanobiological framework in which the physiological responses to static stretching may involve multiple interacting processes rather than a single cellular mechanism. However, direct human evidence linking activation of these cellular pathways to stretching-induced ROM or clinical outcomes remains limited. The following sections examine the principal mechanobiological pathways through which mechanical loading may influence endothelial function, inflammatory activity, extracellular matrix remodeling, and tissue repair.

**Figure 2:**
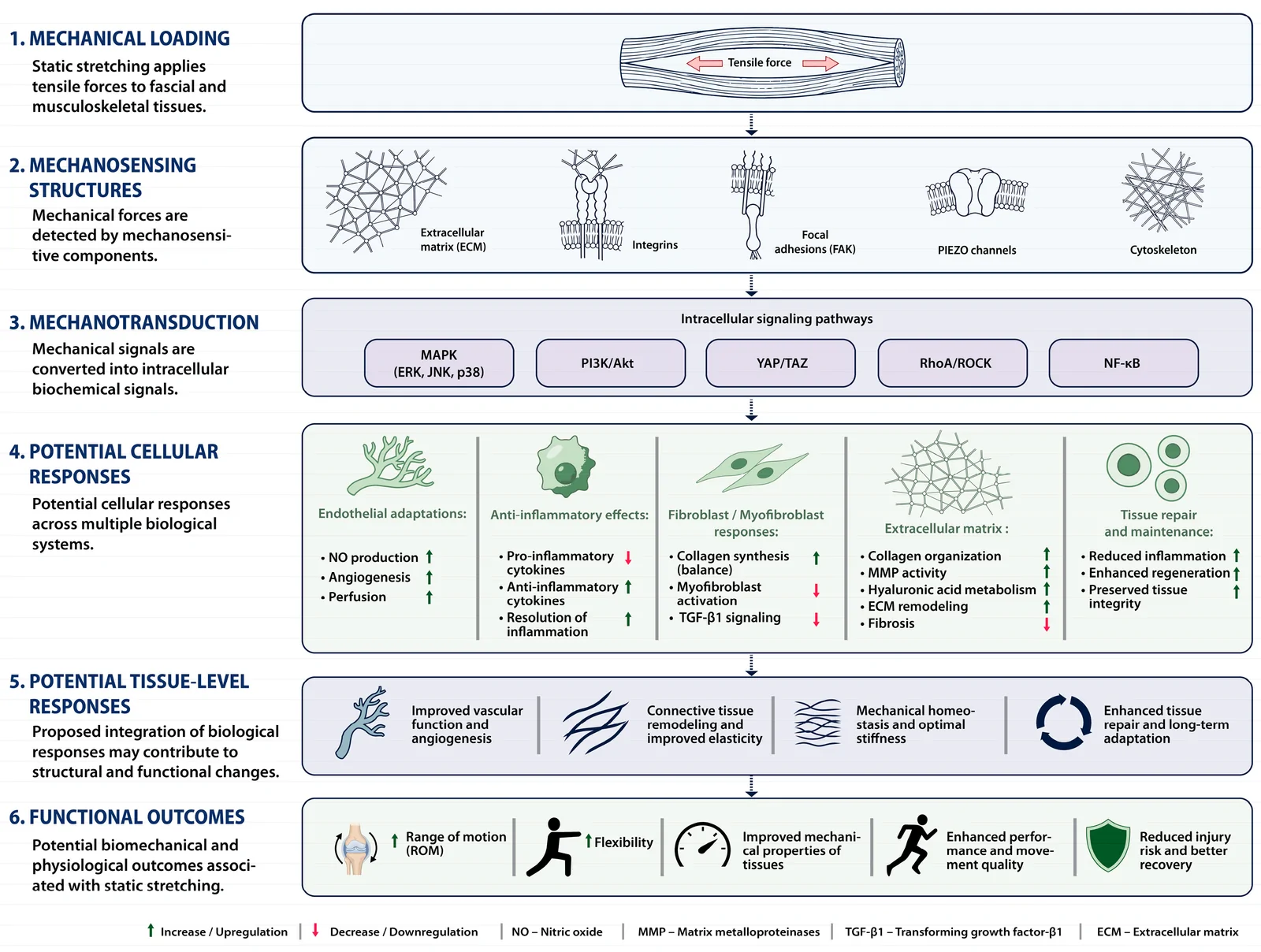
Proposed mechanobiological framework linking mechanical loading during static stretching to cellular, tissue-level, and functional responses. Dashed arrows indicate proposed mechanistic relationships rather than established causal pathways. Supporting evidence varies across the framework and includes direct human static-stretching evidence, indirect human evidence, and preclinical or general mechanobiological evidence.

The mechanobiological interactions discussed throughout the following sections are summarized as a proposed conceptual framework in Figure 2.

Tensile loading generated during static stretching may be sensed through mechanosensitive structures, including the extracellular matrix, integrins, focal adhesions, PIEZO ion channels, and the cytoskeleton. Through mechanotransduction, these mechanical signals can engage intracellular pathways involved in the regulation of endothelial function, inflammatory responses, extracellular matrix remodeling, fibroblast activity, and tissue repair. However, the coordinated activation of these pathways during conventional static stretching and their contribution to long-term musculoskeletal adaptation have not been directly established in humans.

### 5.3. Vascular Mechanobiology

Current mechanobiological evidence identifies endothelial cells as one of the most mechanosensitive cell populations, capable of translating mechanical stimuli into vascular responses through mechanotransduction. Mechanotransduction within endothelial cells contributes to the regulation of vascular homeostasis, endothelial function, and vascular remodeling, providing a general mechanobiological basis for interactions between local mechanical forces and vascular responses [35,39].

Human evidence indicates that repeated passive static stretching can improve vascular function and reduce arterial stiffness [40]. In a 12-week passive static stretching intervention, Bisconti et al. reported improvements in flow-mediated dilation and vascular remodeling, together with reductions in arterial stiffness, in both arteries supplying the stretched muscles and uninvolved arteries [41]. These findings provide direct human evidence of vascular adaptation to repeated stretching, but they do not demonstrate local fascial or extracellular-matrix remodeling. At the molecular level, evidence remains predominantly preclinical: in aged rats, repeated passive splint-induced stretching increased endothelial nitric oxide synthase (eNOS) expression and enhanced endothelium-dependent vasodilation [42]. Thus, although vascular adaptations to stretching are supported by human evidence [40,41], direct demonstration of the proposed molecular mechanisms during conventional static stretching in humans remains limited.

Preclinical evidence suggests that repeated passive stretching can promote angiogenic signaling and structural vascular remodeling. In aged rats, passive splint-induced stretching performed for 30 min/day, 5 days/week for 4 weeks was associated with increased capillarity, microvascular volume and connectivity, together with changes in angiogenesis-related signaling [42]. These findings demonstrate that repeated mechanical stretching can induce microvascular remodeling under this experimental protocol; however, they should not be extrapolated directly to conventional voluntary static stretching in humans. Whether comparable angiogenic or microvascular structural adaptations occur following chronic static stretching in humans remains insufficiently established.

Beyond its vascular effects, endothelial activation is increasingly recognized as a key mediator of communication between the vascular system and surrounding connective tissues. Endothelial-derived signaling molecules influence fibroblast activity, extracellular matrix remodeling, and inflammatory regulation, supporting potential interactions between vascular and connective tissue responses within a broader mechanobiological framework [35,36]. Consequently, angiogenesis may represent one component of this proposed integrated response; however, direct evidence that stretching-induced vascular adaptations mediate local fibroblast activity or extracellular matrix remodeling in humans remains limited.

Collectively, current evidence supports vascular adaptation as one potential mechanobiological pathway linking repeated stretching to broader tissue responses. Human studies provide evidence of changes in vascular function following repeated passive static stretching, whereas evidence for angiogenic and structural microvascular remodeling derives predominantly from preclinical models [40,41,42]. Although improved microvascular function and tissue perfusion could provide a favorable environment for extracellular matrix remodeling and tissue repair, direct evidence that these vascular adaptations mediate long-term connective-tissue or functional adaptations following chronic static stretching in humans remains limited.

### 5.4. Inflammatory Mechanobiology

Current mechanobiological research indicates that mechanical loading regulates not only tissue mechanics but also inflammatory signaling through mechanotransduction. By influencing multiple intracellular pathways involved in the initiation, regulation, and resolution of inflammation, mechanical stimuli contribute to tissue homeostasis and repair. Accordingly, static stretching may be considered not merely a passive mechanical intervention but a biologically active stimulus that may influence the inflammatory microenvironment of mechanically loaded tissues [35,39].

Experimental and translational studies have shown that repeated stretching can modulate the expression of several pro-inflammatory mediators, including nuclear factor kappa B (NF-κB), tumor necrosis factor-α (TNF-α), cyclooxygenase-2 (COX-2), and matrix metalloproteinases involved in extracellular matrix remodeling, including MMP-1, MMP-9, and MMP-13 [43]. However, in vivo rodent evidence indicates that inflammatory responses to stretching can be pro-inflammatory, anti-inflammatory, or mixed depending on the tissue, presence of injury or inflammation, and the duration, frequency, and intensity of the stretching protocol [44]. Simultaneously, in experimental models, stretching can promote anti-inflammatory and pro-resolving responses that facilitate tissue repair and the resolution of inflammation [43,44]. Together, these coordinated molecular responses indicate that the biological effects of mechanical loading extend beyond reductions in passive mechanical stiffness by potentially influencing the restoration of tissue homeostasis [43,44].

Evidence from experimental animal models further supports the anti-inflammatory role of stretching. In a rat model of carrageenan-induced acute subcutaneous connective-tissue inflammation, connective-tissue stretching reduced inflammatory lesion thickness and neutrophil infiltration and increased the pro-resolving mediator resolvin D1; complementary ex vivo experiments similarly showed reduced neutrophil migration and increased resolvin D1 [45]. In a mouse model of connective-tissue injury, brief static tissue stretch (20–30% strain for 10 min twice daily for 7 days) attenuated the injury-associated increase in type-I procollagen, while ex vivo 20% tissue stretch for 10 min reduced soluble TGF-β1 [46]. These findings indicate that mechanical stimulation can influence both inflammatory and profibrotic responses within connective tissue, thereby supporting a potential role in tissue repair [45,46]. However, both studies used preclinical inflammatory or injury models, and their findings cannot be directly extrapolated to conventional static stretching in healthy humans [45,46].

Inflammatory regulation is closely integrated with other mechanobiological processes associated with mechanical loading, including endothelial adaptations, extracellular matrix remodeling, and fibroblast activity. Consequently, modulation of inflammation may be regarded as an integral component of the coordinated adaptive response to mechanical loading rather than an isolated biological effect. The interaction between inflammatory signaling and extracellular matrix remodeling may therefore contribute to long-term structural adaptation; however, its role in adaptations induced by chronic static stretching in healthy humans remains insufficiently established [35,43,44].

Collectively, current evidence indicates that mechanical stretching can modulate inflammatory responses through multiple mechanosensitive signaling pathways rather than a single molecular mechanism. However, evidence for these mechanisms derives predominantly from cellular and preclinical animal models, including tissues with experimentally induced inflammation or injury, and the direction of the response depends on the tissue context and stretching dose [43,44,45,46]. Direct evidence that conventional static stretching produces comparable anti-inflammatory responses in healthy humans remains limited. Thus, inflammatory modulation represents a biologically plausible component of the proposed mechanobiological framework, but its contribution to long-term musculoskeletal adaptation in humans remains uncertain [35,43,44,45,46].

### 5.5. Extracellular Matrix Mechanobiology

Current evidence indicates that extracellular matrix (ECM) remodeling is one of the principal biological processes associated with mechanotransduction in mechanically loaded tissues. Rather than functioning solely as a passive structural scaffold, the ECM represents a dynamic mechanosensitive network that continuously adapts its composition, organization, and mechanical properties in response to externally applied forces. Contemporary mechanobiological models further propose that these adaptations are mediated by reciprocal interactions between fibroblasts and the surrounding matrix, creating a dynamic feedback system that continuously adjusts tissue architecture to changing mechanical demands [47]. Through coordinated interactions among fibroblasts and extracellular matrix components, including collagen fibers and proteoglycans, this process maintains connective tissue integrity while supporting long-term mechanical adaptation [36,39].

Mechanical loading regulates multiple pathways involved in extracellular matrix turnover, including collagen synthesis and degradation, matrix metalloproteinase activity, hyaluronic acid metabolism, and fibroblast activity. Together, these coordinated responses contribute to continuous matrix renewal while preserving tissue integrity and mechanical competence. Recent ex vivo evidence further indicates that collagen degradation is sensitive to both the type and magnitude of applied strain [48]. Importantly, the extent of remodeling depends on the magnitude, duration, and frequency of the applied mechanical stimulus, indicating that connective tissue adaptation is highly dependent on the characteristics of mechanical loading [35,36].

Experimental studies further indicate that stretching can attenuate fibrotic remodeling in preclinical injury and contracture models. In mouse subcutaneous connective tissue, repeated tissue stretching attenuated the injury-associated increase in type-I procollagen [46], whereas in a rat model of posttraumatic knee contracture, static progressive stretching reduced collagen deposition and myofibroblast-related fibrotic changes, with the response depending on stretching duration [49]. These adaptations are accompanied by reduced connective tissue fibrosis and improved joint mobility in the contracture model, indicating that mechanical loading may influence extracellular matrix remodeling and help preserve tissue architecture [46,49]. However, these findings derive from pathological animal models and do not establish comparable antifibrotic ECM adaptations following conventional static stretching in healthy humans [46,49].

Beyond its structural role, the extracellular matrix functions as an active mediator of cellular behavior by transmitting mechanical information between neighboring cells and the surrounding tissue environment. Dynamic interactions between fibroblasts and the ECM enable connective tissues to continuously adjust their mechanical properties according to functional demands, thereby linking mechanotransduction with long-term tissue adaptation. This reciprocal relationship between cells and the extracellular matrix is increasingly recognized as a key mechanism underlying connective tissue plasticity and adaptation [35,36].

Collectively, current evidence indicates that extracellular matrix remodeling represents a fundamental biological mechanism through which mechanical loading may influence connective tissue adaptation. By regulating collagen turnover, fibroblast activity, matrix organization, and antifibrotic signaling, mechanotransduction provides a molecular framework for the long-term structural adaptations associated with repeated mechanical loading [35,36,39]. Despite these advances, most mechanobiological evidence for structural ECM remodeling derives predominantly from cell culture and animal models, whereas direct human evidence is limited to acute responses and does not establish sustained local ECM remodeling following chronic static stretching [50]. Thus, ECM remodeling represents a biologically plausible component of adaptation to repeated static stretching, but its contribution to long-term connective-tissue adaptation in healthy humans remains uncertain. The experimental models and directness of evidence supporting the proposed ECM effects are summarized in Supplementary Table S3. Additional in vivo studies are therefore needed to clarify the temporal sequence, magnitude, and clinical significance of extracellular matrix remodeling following chronic static stretching [35,39].

### 5.6. Mechanotransduction as an Interface Within the Proposed Integrative Framework

The evidence presented throughout this section supports mechanotransduction as an important biological interface through which mechanical loading may influence cellular and tissue-level responses during static stretching. Rather than acting through a single biological pathway, stretching may engage multiple, interacting mechanosensitive processes involving endothelial cells, fibroblasts, myofibroblasts, skeletal muscle fibers, and fascial tissues. Together, these responses may influence vascular function, inflammatory signaling, extracellular matrix remodeling, and tissue repair, thereby contributing to the maintenance of structural integrity and mechanical homeostasis [35,39].

Importantly, the biological effects of static stretching may emerge from interactions among these adaptive processes rather than from isolated cellular responses. Vascular adaptations may improve tissue perfusion, inflammatory regulation may support tissue repair, and extracellular matrix remodeling may contribute to the structural and mechanical adaptation of connective tissues. These processes can occur in parallel and interact across multiple biological levels, with mechanotransduction providing an important interface between mechanical loading and cellular and tissue-level responses [35,39].

Current evidence therefore suggests that mechanotransduction may provide a common biological framework for understanding many of the structural and physiological adaptations associated with chronic static stretching. This perspective is consistent with recent evidence indicating that stretching-induced adaptations involve interactions among mechanical, neural, and tissue-specific mechanisms rather than reflecting a single isolated physiological process [3]. Direct human evidence further demonstrates that neural and mechanical responses can occur concurrently during and immediately after acute static stretching, although such acute responses do not by themselves establish stable neural or structural adaptations following chronic stretching [20].

Nevertheless, mechanotransduction alone cannot fully explain the functional adaptations associated with chronic stretching. Although connective tissue remodeling provides an important biological foundation for long-term adaptation, accumulating evidence indicates that changes in stretch tolerance, sensory perception, and neural regulation also contribute to increases in joint range of motion. These neural and perceptual responses should not be regarded simply as downstream consequences of cellular mechanotransduction; rather, neural, perceptual, mechanical, and tissue-level processes may occur in parallel and interact during the response and adaptation to stretching [3,20]. A comprehensive understanding of static stretching therefore requires integration of tissue-level mechanobiological adaptations with neural regulatory mechanisms.

Collectively, the evidence presented in this chapter supports the concept of mechanotransduction as an important biological interface between externally applied mechanical forces and tissue-level responses and adaptations. Rather than representing an isolated cellular process, mechanotransduction forms part of an interacting system involving mechanical, vascular, inflammatory, extracellular-matrix, neural, and perceptual processes. However, the directness and temporal scale of evidence differ substantially across these mechanisms: direct human evidence is available for both acute and chronic mechanical and vascular responses, whereas evidence for neural responses is stronger for acute effects and remains less consistent for stable chronic adaptation. In contrast, evidence for sustained cellular, inflammatory, and extracellular-matrix remodeling following chronic static stretching remains predominantly indirect or preclinical. Accordingly, the proposed framework should be regarded as an integrative mechanobiological model rather than a demonstrated linear causal pathway. This integrated perspective provides the conceptual foundation for the neural mechanisms discussed in the following chapter.

## 6. Neural Adaptations

### 6.1. Sensory and Mechanical Contributions to ROM Improvements

Increased joint range of motion (ROM) is the most consistent adaptation reported following both acute and chronic static stretching interventions [3,6]. Improvements have been demonstrated across different muscle groups, populations, and stretching protocols, making enhanced flexibility the most reproducible long-term outcome associated with static stretching [1,3]. However, the mechanisms responsible for these improvements have been fundamentally reinterpreted over the past two decades.

Traditionally, increases in ROM were attributed to permanent elongation of skeletal muscle or other components of the muscle–tendon unit. This interpretation assumed that repeated stretching progressively altered tissue structure and reduced passive stiffness. However, substantial improvements in ROM can occur after only a few stretching sessions—or even after a single bout—an adaptation that is unlikely to reflect meaningful morphological remodeling of muscle or connective tissue within such a short time frame [3,8].

These rapid improvements are unlikely to reflect meaningful structural tissue remodeling and may instead involve changes in stretch tolerance together with acute alterations in passive mechanical properties. Stretch tolerance refers to the ability to tolerate greater passive tension or discomfort before terminating the stretch without requiring a proportional increase in tissue extensibility. Accordingly, early increases in ROM may partly reflect altered sensory processing and changes in stretch tolerance, alongside acute mechanical changes [3,8]. Importantly, however, the recent meta-analysis by Ingram et al. found an overall increase in maximum tolerable passive resistive torque following chronic, but not acute, static stretching, indicating that increased stretch tolerance should not be assumed to be the predominant mechanism underlying immediate ROM gains [3].

This concept was formalized by Weppler and Magnusson, who proposed that many stretching-induced improvements in flexibility are better explained by changes in tolerance to stretch-related discomfort than by lasting alterations in the viscoelastic properties of the muscle–tendon unit [8]. Their work fundamentally shifted the interpretation of flexibility adaptation from structural tissue elongation toward neural and sensory regulation of the stretching endpoint.

Subsequent evidence has generally supported this interpretation. Freitas et al. reported that stretching interventions lasting up to approximately eight weeks reliably increased ROM, whereas evidence for structural adaptations within the muscle–tendon unit remained limited and inconsistent [51]. Similarly, Behm et al. concluded that improvements in flexibility often exceed the magnitude of measurable mechanical changes, indicating that neural mechanisms contribute substantially to the early response to stretching [6]. Collectively, these findings suggest that increases in ROM frequently precede detectable remodeling of muscle architecture or connective tissue, supporting the potential contribution of sensory mechanisms while not excluding concurrent acute changes in passive mechanical properties.

This distinction has important implications for the interpretation of stretching studies. Increases in ROM should not automatically be interpreted as evidence of greater tissue extensibility or reduced passive stiffness, particularly following acute or short-term stretching interventions. Instead, early improvements may reflect contributions from altered sensory processing together with acute changes in passive mechanical properties, with their relative contributions remaining incompletely resolved [3,8].

Collectively, current evidence indicates that acute and chronic improvements in ROM may involve different and potentially overlapping mechanisms. Acute ROM gains may involve sensory changes together with alterations in passive mechanical properties, whereas increased stretch tolerance appears more consistently supported following chronic static stretching [3]. With prolonged interventions, modest reductions in passive stiffness may also contribute to these adaptations, suggesting that chronic gains in flexibility may reflect combined sensory and mechanical mechanisms rather than a single physiological process [3].

### 6.2. Central Nervous System Adaptations

Although increased stretch tolerance is widely recognized as an important mechanism underlying early improvements in joint range of motion (ROM), the neurophysiological processes responsible for this adaptation remain incompletely understood. Current evidence indicates that these improvements cannot be attributed to a single neural mechanism but may involve contributions from peripheral sensory input, spinal reflex modulation, and central nervous system (CNS) processing and regulation [6,52].

One influential explanation proposes that repeated stretching progressively alters the central perception of stretch-induced discomfort rather than the mechanical limits of the musculoskeletal tissues. Weppler and Magnusson suggested that repeated stretching increases the threshold at which stretch-related discomfort becomes intolerable, thereby allowing individuals to tolerate greater passive elongation without substantial changes in tissue mechanical properties [8]. Consequently, considerable improvements in ROM may occur despite only minimal alterations in the structural properties of the muscle–tendon unit.

Altered processing of nociceptive and mechanosensory information has been proposed as a contributor to this phenomenon. Static stretching provides sensory input from mechanosensitive and nociceptive afferents associated with muscles, fascia, tendons, joint capsules, and other connective tissues. Repeated exposure to these sensory inputs may alter the perception and processing of stretch-related sensations, reducing the perception of stretch-related discomfort without necessarily altering the mechanical properties of the tissues. Indirect support for a sensory contribution is provided by the systematic review of Freitas et al., who reported consistent increases in ROM following stretching interventions lasting up to approximately eight weeks despite limited evidence of structural adaptations within the muscle–tendon unit [51].

Several neurophysiological mechanisms have been proposed to explain these adaptations. At the spinal level, repeated stretching has been proposed to attenuate reflex-mediated protective muscle activity, thereby potentially reducing involuntary resistance to passive elongation. At the supraspinal level, adaptations have been proposed to involve changes in sensory integration, motor planning, and the cognitive evaluation of stretch-related sensations. Collectively, these neurophysiological processes could contribute to reduced protective muscle guarding, allowing greater joint excursions without substantial alterations in the passive mechanical behavior of the muscle–tendon–fascia complex [6,52].

Despite these plausible mechanisms, direct neurophysiological evidence remains limited. Shah et al. concluded that although static stretching consistently increases ROM, persistent changes in reflex excitability, corticospinal excitability, and neuromuscular activation have not been demonstrated consistently across studies [52]. Similarly, Konrad et al. confirmed the robust effect of chronic stretching on ROM but emphasized that the available evidence does not allow these improvements to be unequivocally attributed to specific neural adaptations because of methodological heterogeneity and the limited availability of direct neurophysiological measurements [1].

More recently, Ingram et al. further refined this interpretation by reporting that improvements in ROM following chronic static stretching are associated with both increased stretch tolerance and reduced passive stiffness [3]. However, the authors also emphasized that the neurophysiological basis of increased stretch tolerance remains unresolved, underscoring the need for future studies integrating biomechanical, neurophysiological, and mechanobiological approaches.

Collectively, current evidence indicates that early improvements in ROM are strongly associated with increased stretch tolerance and may involve changes in sensory perception, while the contribution of specific central neural mechanisms remains uncertain. Nevertheless, the relative contributions of central nervous system modulation, altered nociceptive processing, spinal reflex adaptation, and supraspinal regulation remain incompletely understood. Direct evidence for stable neurophysiological adaptations remains limited, and mechanical changes may also contribute, particularly with repeated stretching [3,52]. Future studies integrating biomechanical assessments with advanced neurophysiological techniques will be essential to clarify how these interacting neural mechanisms contribute to flexibility adaptations following static stretching.

### 6.3. Electromyographic Evidence

Electromyography (EMG) is one of the principal methods used to investigate neuromuscular responses and adaptations associated with static stretching. By providing an indirect measure of muscle electrical activity, EMG has been widely used to examine whether changes in neural activation accompany the consistent increases in joint range of motion (ROM) observed following stretching interventions.

Acute evidence indicates that muscle activity may be reduced during or immediately after passive stretching, suggesting transient changes in neural activation and reflex-mediated muscle activity [13]. These findings have been interpreted as evidence that stretching may transiently reduce neural resistance to passive elongation, thereby permitting greater joint excursions. Likewise, reductions in spinal reflex excitability and alterations in motor output have been proposed as potential mechanisms contributing to increased stretch tolerance [52].

However, the available neuromuscular evidence remains inconsistent. Although some studies have reported reductions in muscle activity or reflex excitability following static stretching, others have found minimal or no significant changes in neuromuscular activation after either acute or chronic interventions. This inconsistency may partly reflect methodological heterogeneity, including differences in stretching protocols, intervention duration, muscle groups examined, EMG normalization procedures, contraction intensity, and participant characteristics, thereby limiting direct comparisons across studies [52].

Recent evidence further indicates that EMG findings alone cannot account for the magnitude of flexibility gains observed following static stretching. Although changes in neuromuscular activation may contribute to increased stretch tolerance, direct evidence for persistent alterations in corticospinal excitability, spinal reflex excitability, or neuromuscular activation remains limited [52]. Likewise, Konrad et al. confirmed that chronic stretching consistently increases ROM across different populations and intervention protocols but concluded that the underlying mechanisms remain incompletely understood [1]. Collectively, these findings indicate that changes in muscle activation represent only one component of a broader adaptive response.

Collectively, current evidence suggests that EMG should be regarded as a complementary rather than definitive indicator of the neuromuscular responses and adaptations associated with static stretching. Reduced muscle activity and altered motor output may contribute to reduced resistance to passive elongation and facilitate greater ROM but are insufficient to explain the full magnitude of flexibility improvements. Instead, increases in ROM are likely to reflect contributions from multiple mechanisms, including neural regulation, altered sensory perception, and mechanical adaptations within the muscle–tendon–fascia system [1,3,52].

### 6.4. Sensory Integration Within the Mechanobiological Framework

Although stretch tolerance, central modulation, and electromyographic responses are often discussed as distinct mechanisms, they may represent interacting components of a broader sensorimotor regulatory system [20]. Mechanical deformation induced by static stretching may generate sensory input not only through muscle spindles and Golgi tendon organs but also through mechanosensitive receptors distributed throughout fascial tissues, including Ruffini endings, Pacinian corpuscles, and free nerve endings [16,25]. Consequently, sensory information generated during stretching may originate from multiple mechanically interconnected tissues rather than from skeletal muscle alone, contributing to afferent information available to the central nervous system (CNS) regarding tissue loading and body position [16,25].

The fascial network has been proposed to function not only as a mechanical force-transmission system [26] but also as an important sensory structure potentially contributing to proprioception, interoception, and nociception [16,25]. Mechanical stimulation of fascial mechanoreceptors may contribute to afferent input through peripheral sensory pathways and thereby potentially influence spinal reflex excitability, supraspinal processing, and motor output [16,25]. Such integration of mechanical and sensory information may contribute to CNS regulation of muscle activation in response to tissue loading; however, these relationships have not been directly established during conventional static stretching in humans.

In addition to proprioceptive regulation, stretching-induced sensory input may also influence pain perception through modulation of nociceptive signaling. Activation of low-threshold mechanoreceptors, together with changes in local tissue perfusion and inflammatory mediator activity, has been proposed to contribute to reduced nociceptive drive and altered central pain processing [16,25,44]. These interacting neurophysiological mechanisms may provide one possible explanation for changes in pain perception associated with stretching; however, their specific contribution and the underlying pathways remain uncertain.

Collectively, current evidence suggests that neural adaptation to static stretching extends beyond isolated changes in reflex excitability or muscle activation. Sensory integration represents one possible link between peripheral mechanical loading and changes in stretch tolerance, although its relative contribution remains uncertain. Rather than constituting a central mechanism, sensory processes may interact with fascial mechanics, mechanotransduction, and motor control within the broader response to stretching. This integrative sensorimotor perspective provides a conceptual bridge between the cellular mechanisms described in the previous chapter and the systems-level neural regulation incorporated into the unified mechanobiological framework proposed in the concluding chapter.

## 7. Integrative Mechanobiological Model

### 7.1. From Mechanical Loading to Biological Adaptation

The evidence synthesized throughout the preceding chapters supports a progressive reinterpretation of static stretching as a multilevel mechanobiological process rather than a simple mechanical intervention. Instead of producing isolated effects within individual tissues, externally applied tensile loading may engage multiple interacting processes across different levels of biological organization. These processes include force transmission through the myofascial system, cellular mechanosensing and mechanotransduction, connective-tissue responses, and changes in neural regulation, which may occur in parallel and interact rather than forming an established sequential cascade [10,22,25,51,52]. Their temporal ordering and causal relationships remain incompletely established, particularly because evidence for several cellular and tissue-level mechanisms remains indirect or preclinical.

Within this framework, the myofascial system represents an important interface through which externally applied forces may be distributed across mechanically interconnected tissues. Owing to its continuous three-dimensional organization, fascia may contribute to the transmission of tensile loading among interconnected muscles, tendons, ligaments, peripheral nerves, and surrounding connective tissues, creating an integrated biomechanical environment in which mechanical deformation may generate sensory and mechanobiological responses across multiple tissue structures [16,22,25,26]. Static stretching should therefore be regarded not as loading an isolated anatomical structure but as applying mechanical loading within an interconnected biological system [16,22,25,26].

Mechanical deformation may be detected by specialized mechanosensitive structures, including integrins, focal adhesion complexes, mechanosensitive ion channels, and the cytoskeleton [11,35,36,37]. Through mechanotransduction, these structures can convert mechanical stimuli into intracellular biochemical signals involved in the regulation of gene expression, protein synthesis, extracellular matrix turnover, endothelial function, inflammatory activity, and fibroblast behavior [35,36,39,47]. These cellular processes may interact across multiple biological levels rather than occurring in an established temporal sequence. However, much of the evidence supporting these pathways derives from general mechanobiological, cellular, or preclinical models, and their specific contribution to adaptation following conventional static stretching in humans remains incompletely established.

At the tissue level, mechanical loading may be associated with extracellular matrix remodeling, changes in collagen organization, regulation of vascular function, and modulation of inflammatory signaling [39,42,43]. In parallel, neural mechanisms may contribute to changes in stretch perception, sensorimotor integration, and regulation of muscle activity [51,52]. These tissue-level and neural processes may interact across multiple levels of biological organization rather than occurring as a fixed sequential cascade; however, their temporal relationships and causal contributions to chronic static-stretching adaptation remain incompletely established. Evidence for vascular adaptation includes preclinical stretching models, whereas evidence for sustained extracellular-matrix, inflammatory, and neural adaptations remains heterogeneous and, for several mechanisms, indirect [39,42,43,51,52].

Rather than representing independent physiological phenomena, myofascial force transmission, mechanotransduction, extracellular matrix remodeling, and neural modulation may be viewed as interacting components of a proposed multilevel adaptive network [16,22,39,51]. These processes may occur in parallel or interact recursively, and their temporal ordering and causal relationships have not been established. This integrative perspective provides the conceptual foundation for the mechanobiological model proposed in the present review.

### 7.2. A Proposed Integrative Mechanobiological Framework for Static Stretching

Current evidence no longer supports the interpretation of static stretching as an intervention acting through a single tissue or physiological mechanism. Instead, the findings synthesized throughout this review support a conceptual framework in which its effects may involve multiple mechanically and biologically interconnected processes. Myofascial force transmission, cellular mechanotransduction, extracellular matrix remodeling, vascular regulation, and neural adaptation may therefore be regarded as interacting components of a proposed multilevel adaptive network rather than as elements of an established sequential pathway.

**Figure 3:**
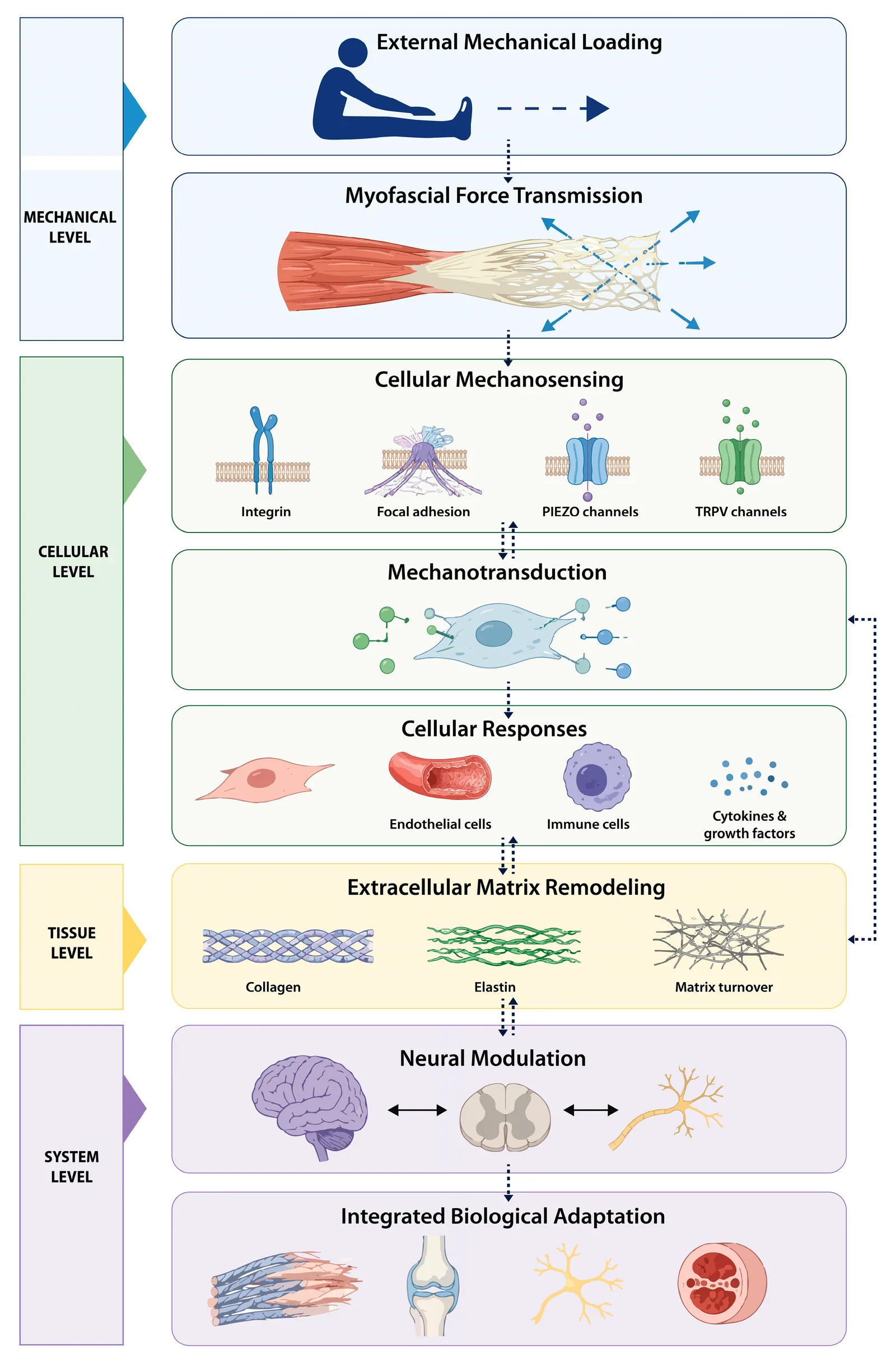
Conceptual model illustrating the proposed integrative mechanobiological framework of static stretching. Dashed arrows indicate proposed interactions between levels of biological organization and do not imply an established sequential or causal pathway. Bidirectional and lateral dashed connections illustrate potential reciprocal and cross-level interactions. Processes may occur in parallel or interact recursively, and their temporal ordering remains incompletely established.

The proposed conceptual framework is summarized in Figure 3, which illustrates how externally applied tensile forces may engage interacting adaptive processes across multiple levels of biological organization.

Figure 3 illustrates the conceptual model proposed in the present review, describing potential interactions between externally applied mechanical loading and biological responses across multiple levels of biological organization. Mechanical forces may be distributed throughout the myofascial continuum and may be detected by mechanosensing cellular structures, while mechanotransduction may generate intracellular biochemical signals that interact with processes involved in extracellular matrix remodeling and neural modulation. As indicated by the bidirectional and lateral dashed connections, interactions among these processes may extend across levels of biological organization and may occur in parallel or recursively rather than constituting an established sequential pathway.

The proposed model emphasizes that these responses should not be interpreted as independent physiological phenomena. Instead, mechanical force transmission, cellular signaling, extracellular matrix remodeling, vascular adaptation, inflammatory regulation, and neural modulation may interact as components of a proposed integrative mechanobiological network. The biological effects of static stretching may therefore reflect interactions among these processes rather than the action of any single adaptive mechanism.

An important implication of this framework is that it generates a testable hypothesis regarding inter-individual variability in responses to static stretching, which has been reported across the literature and directly observed in tissue-mechanical responses [1,3,53]. Individual differences in connective-tissue composition, fascial properties, mechanosensitive signaling capacity, neural regulation, and loading history may represent candidate moderators of these adaptive responses. However, the contribution of these factors to inter-individual variability remains hypothetical and requires direct moderator and mediation studies. Static stretching may therefore be considered a context-dependent mechanobiological intervention whose effects may reflect interactions between tissue-specific mechanical properties and biological responsiveness rather than mechanical loading alone.

### 7.3. Conceptual Implications

The integrative mechanobiological framework proposed in this review extends the traditional interpretation of static stretching beyond isolated physiological mechanisms. Classical explanations have primarily emphasized increased muscle extensibility, reduced passive stiffness, altered stretch tolerance, or changes in neural activity [1,8]. Although each of these mechanisms is supported by available evidence, no single mechanism appears sufficient to account for the broad spectrum of structural and physiological adaptations observed following repeated stretching. Instead, current evidence supports considering these adaptations within a framework of potentially interacting biological processes rather than as isolated responses within individual tissues [3,12].

From this perspective, static stretching should no longer be regarded as an intervention targeting a single anatomical structure. Rather, it may be conceptualized as a multilevel mechanobiological stimulus acting across mechanically interconnected tissues and biologically integrated regulatory systems. Mechanical loading may be distributed throughout the myofascial continuum and may engage mechanosensing and mechanotransduction processes that interact with extracellular matrix remodeling, vascular responses, and neural regulatory mechanisms; these processes may occur in parallel or interact recursively rather than forming an established sequential cascade [9,10,12,39]. However, several of these proposed interactions are supported primarily by general mechanobiological or indirect evidence, and their specific contribution to conventional static stretching in humans remains incompletely established. Consequently, the biological response may reflect interactions among these processes rather than the isolated adaptation of muscles, fascia, connective tissues, or the nervous system.

The proposed framework also generates a testable hypothesis regarding inter-individual variability reported in the stretching literature [1,3,53]. Individual differences in connective tissue composition, fascial mechanical properties, extracellular matrix organization, mechanosensitive signaling capacity, neural regulation, and loading history may represent candidate moderators of how mechanical stimuli are perceived and translated into biological responses [19,24,32,33,35,39]. However, these factors have not been established as moderators of individual responses to static stretching and require direct moderator and mediation studies. Consequently, identical stretching protocols may produce markedly different adaptations despite comparable external loading conditions. This perspective raises the possibility of tissue-specific and individual-specific mechanobiological adaptation rather than establishing such adaptation as a demonstrated mechanism.

The proposed framework also identifies several priorities for future research. Although considerable progress has been made in understanding the mechanobiology of connective tissues, important questions remain regarding the temporal interactions among mechanotransduction, extracellular matrix remodeling, endothelial adaptation, inflammatory regulation, and neural modulation, particularly in humans [35,36,39]. Future investigations integrating advanced imaging techniques, molecular biomarkers, tissue biomechanics, and neurophysiological assessments will be essential for clarifying whether and how these mechanisms interact over time and contribute to long-term adaptations to static stretching. Such integrative approaches may also facilitate the development of individualized stretching interventions based on tissue-specific mechanical characteristics and biological responsiveness.

It is important to acknowledge that the framework presented in this review represents a conceptual synthesis of the currently available evidence rather than a definitive mechanistic model. Much of the mechanobiological evidence originates from cell culture studies, animal models, or investigations of individual physiological systems, whereas direct human evidence integrating mechanical, cellular, extracellular, vascular, and neural adaptations remains comparatively limited [9,35,39]. The literature screening and evidence classification were performed by a single reviewer, and no formal risk-of-bias assessment was undertaken. Therefore, despite the structured search and evidence-directness classification, the possibility of study-selection, classification, and interpretative bias cannot be excluded. Consequently, the proposed framework should be viewed as a conceptual model that integrates current knowledge while providing a theoretical basis for future experimental testing, validation, and refinement.

Collectively, this integrative perspective extends the interpretation of static stretching beyond isolated tissue-specific mechanisms. By unifying myofascial force transmission, mechanotransduction, extracellular matrix remodeling, vascular regulation, and neural adaptation within a single conceptual model, it provides an integrative framework for examining how externally applied mechanical loading may be associated with interacting adaptive processes across multiple levels of biological organization, without implying an established sequential or causal pathway. This perspective may guide future mechanobiological research and support the development of more biologically informed approaches to stretching in sports medicine, rehabilitation, and exercise science [3,12].

## 8. Conclusions

Static stretching has traditionally been interpreted as a flexibility intervention acting primarily through increases in muscle extensibility and reductions in passive tissue stiffness. However, the evidence synthesized throughout the present review suggests that this classical muscle-centered paradigm may be insufficient to account for the broader biological responses associated with repeated mechanical loading. The strongest conclusion supported by the current evidence is that static stretching is unlikely to be adequately characterized by a single-tissue or single-mechanism model.

The integrative mechanobiological framework proposed in this review suggests that externally applied mechanical loading may be linked to biological adaptation through a network of potentially interacting processes involving myofascial force transmission, cellular mechanotransduction, extracellular matrix remodeling, vascular regulation, and neural modulation. Rather than representing a proven sequential cascade or a unified adaptive system, these processes are proposed to interact in parallel, reciprocally, or recursively, while their relative contributions and causal integration in human static stretching remain incompletely established.

This perspective provides a conceptual basis for interpreting the heterogeneous findings reported in the stretching literature and generates a testable hypothesis regarding the substantial inter-individual variability observed following similar stretching interventions. It further proposes that individual responses to static stretching may reflect interactions between tissue-specific mechanical properties and biological responsiveness rather than mechanical loading alone, although the contribution of these candidate moderators requires direct investigation.

Although several mechanobiological pathways remain incompletely understood, particularly in humans, the proposed framework provides a conceptual basis for future investigations aimed at testing the proposed interactions and their relative contributions, while integrating tissue biomechanics, molecular mechanobiology, and neurophysiology. Such multidisciplinary approaches may facilitate the development of more biologically informed, tissue-specific, and individualized stretching interventions in sports medicine, rehabilitation, and exercise science.

Collectively, the integrative mechanobiological framework presented in this review proposes that static stretching may be more appropriately considered within a multilevel mechanobiological perspective rather than a single-tissue or single-mechanism model. By integrating myofascial mechanics, mechanotransduction, extracellular matrix remodeling, vascular regulation, and neural adaptation within a single conceptual model, it provides a coherent framework that identifies candidate mechanobiological links requiring further validation. The relative contributions and causal integration of these processes, however, remain incompletely established in humans.

## Data Availability

No new data were created or analyzed in this study.

## Supplementary Materials

The following supporting information can be downloaded at: https://www.mdpi.com/article/10.3390/jfmk11030365/s1, Supplementary Table S1: Complete Database Search Strategies; Supplementary Table S2: Evidence Map; Supplementary Table S3: Experimental Models and Directness of Evidence for Proposed Extracellular-Matrix Effects of Mechanical Stretching.
